# Self-Reported Study Analyzing Physicians’ Personal Compliance with Health Prevention Guidelines in a Medium-Sized Canadian Community

**DOI:** 10.1177/21501319231162480

**Published:** 2023-03-27

**Authors:** Fawad Ahmed, Ryan Craig, Abeer Omar, Maher El-Masri

**Affiliations:** 1 Western University, London/Windsor, ON, Canada; 2 Trent University, Toronto, ON, Canada; 3 Ryerson University, Toronto, ON, Canada

**Keywords:** physicians, survey, health, screening, preventative, treatment, promotion

## Abstract

There have only been limited studies that have assessed the attitude of Canadian physicians toward their own physical health. The aim of our study was to explore the self-reported health maintenance behavior and the predictors of health practices among physicians in a small-medium sized Canadian community. We used a descriptive mailed in self-report survey to contact all 649 physicians registered with the Essex County Medical Society, with a 36% response rate. Our results showed that 81% of physicians in Windsor-Essex County were satisfied with how well they care for themselves, despite reporting low levels of physical activity and a lower percentage of respondents having family physicians than the general population. Five independent factors were identified with physician self-perceived health satisfaction: Physician age of 45 to 54 (95% CI 0.17-0.92; OR 0.39), graduating from Canadian medical schools (95% CI 0.15 to 0.80; OR 0.35), having more than one co-morbidity (95% CI 0.13-0.72; OR 0.31), physicians who had a regular family doctor (95% CI 1.12-5.52; OR 2.43), and engagement in regular moderate weekly exercise (95% CI 1.05-4.94; OR 2.28). We also contrasted the preventive health screening markers of our study to compliance rates of the general population as well as the national physician study. Our results showed that screening rates among our study physician group differed markedly from the general population. For colorectal and breast cancers, physicians in our study reported screening rates of 77.8% and 37.3% respectively, compared with the general population, who’s screening rates are 32.3% and 72.5%. Future studies exploring specific targeted health promotion interventions that could address these factors may be warranted in order to further improve Canadian physician health, and ultimately improve their ability to take care of their patients.

## Introduction

Research in the last 2 decades has shown an important link between physicians’ personal wellness and the patient care they provide.^1,2^ Physician wellness is a holistic term that refers to different segments of a physician’s health. Specifically, it includes a physician’s physical, emotional, and mental well-being.^3^ Although a plethora of research reports are available on the mental and emotional health of physicians, only a few studies have been conducted about their physical health.^4,5^ Moreover, increasing evidence suggests that physicians are likely to neglect their own physical health.^6^

Many physicians are reluctant to see a family physician for their own care. For instance, a survey of Spanish physicians found that 49% of respondents did not have a family doctor. Of those who did, only 48% reported they followed their primary care physician’s advice.^7^ Studies of Irish and Australian physicians found similar rates of physician indifference to their own health.^3,8,9^ Further, the evidence-based preventive health recommendations have variable rates of compliance among physicians. For example, findings from several studies^10-12^ suggested that mammograms among appropriate-age female physicians ranged between 47% and 81%. Similarly, cholesterol monitoring rates among physicians were also variant and ranged between 52% and 90%.^13^ Moreover, the rate of self-treatment among physicians was 90% for acute conditions and 25% for chronic conditions, despite it being inappropriate given its lack of objectivity.^6^

The purpose of this study was therefore to explore the health maintenance behaviors of Canadian physicians in a small-medium size community by assessing their self-reported responses about their own health. This is important given that there has only been a handful of studies that assessed the attitude of Canadian physicians toward their physical health and health maintenance behaviors.^10^ In fact, such research has not been conducted on physicians practicing in small to medium sized communities, which interestingly make up about one-third of Canadian medical practice.^14^ The data gleaned from this study could have significant implications for promoting better physician health. Given that physicians’ personal health maintenance habits influence their counseling of patients,^15^ improving physician health is likely to positively impact patient care as a whole. Of note, this study was conducted just prior to the onset of the COVID-19 pandemic. The data from this study may also provide a good reference point for future studies looking at how COVID-19 pandemic affected physician health as a whole.

## Methods

Upon approval from the institutional Research Ethics Boards, we conducted a descriptive cross-sectional survey using a self-report questionnaire between January and March 2015 in a southwestern Ontario county that is comprised of 1 city and 7 towns with a total population of 388 000. A printed copy of the survey along with a printed description of the study’s purpose and a pre-paid return envelope were mailed out to a complete list of all 649 local physicians who were registered with the local district of the Ontario Medical Association. Physicians in all specialties were included in the survey.

Questions in our survey were developed for the purpose of this study based on an extensive review of the literature to make sure that we included relevant questions. Furthermore, we referenced specific screening and vaccination guidelines, as well as the Preventive Care Checklist forms that are endorsed by the College of Family Physicians of Canada.^16^ With author permission, many of the questions in our survey were taken verbatim from, or based on, the Canadian Medical Association’s Health Practices of Canadian Physicians study.^10^ This allowed us to contrast findings from a small to medium sized community with the findings reported in a national level data. We sought expert advice from several physicians during the survey development phase, and piloted data collection with 15 physicians to ensure the feasibility of data collection procedures and the clarity of the survey questions. To minimize bias, physicians who participated in the pilot phase were excluded from the study.

In order to maximize the response rate of participants, a written reminder was sent to all 649 physicians after 1 month of sending the original survey. From the original mailing list, 34 physicians could not be reached due to incorrect mailing addresses, thus reducing the study population to 615 potential participants. Responses that were received within 8 weeks of the second reminder mailing were included in our data analysis. Out of the 615 potential participants, 225 responded, yielding a reasonable response rate of 36.6%, which was close to the response rate of 40.5% reported in the national study.^10^

Data were analyzed using the IBM.SPSS software (version 26.0). Basic descriptive statistics were performed to describe the demographic characteristics of the sample, and the frequency of their self-reported lifestyle, and personal health reflections. The variable “self-perceived satisfaction with own health” was dichotomized and analyzed as the primary outcome variable of the study. The categories of the original 5-point Likert scale outcome variable were merged into 2 categories (satisfied = very satisfied and somewhat satisfied, dissatisfied = somewhat dissatisfied and very dissatisfied). Chi-square and Mann-Whitney comparisons were performed to explore the unadjusted differences of the study variables on the outcome variables. Fisher’s exact comparisons were conducted instead of Chi-square for nominal variable comparisons when at least 1 cell in the contingency table had a count of less than 5. Given the exploratory nature of the study, stepwise logistic regression analyses were then conducted to explore the independent predictors of “self-perceived satisfaction with own health.” Independent variables were included in the logistic regression models based on a liberal alpha of ≤.25 in order to maximize the parsimony of the regression model and to avoid the exclusion of potentially important variables.^17^ Statistical significance was established using 95% CI.

## Results

### Sample Characteristics

Tables 1 and 2 shows that the majority of our participants were male (70.2%). While 45.3% were in the age range of 55 to 65 years, 60.4% and 74.4% completed their medical education and residency in Canada, respectively. The responses were almost equally divided between family medicine (n = 105; 46.7%) and specialties (n = 120; 53.3%). Interestingly, the mean BMI of the total sample was 26.3. About one-third of the participants did not have a regular GP, had comorbidities, and their last physical checkup was more than 2 years ago (Table 3). The results indicate that 94.7% of participants thought they were in good to excellent health. However, of those only 81% felt satisfied about taking care of their own health (Table 3).

**Table 1.** Mann-Whitney *U* Comparisons of Sample Characteristics on Physicians’ Self-Perceived Satisfaction With Taking Care of Own Health.

Variable	Dissatisfied (n = 43)	Satisfied (n = 182)	Total (N = 225)	*Z*	*P*
Body Mass Index				−1.91	.057
Mean (SD)	27.7 (5.3)	26.0 (4.6)	26.3 (4.8)		
Median	26.8	25.1	25.5		
Hours/week for professional activities				1.58	.114
Mean (SD)	6.2 (3.7)	7.8 (4.8)	7.5 (4.6)		
Median	6.0	8.0	8.0		
Hours/week on patient care				−1.82	.068
Mean (SD)	45.4 (11.2)	41.5 (12.0)	42.2 (12.0)		
Median	42.0	40.0	40.0		
Practicing years				2.95	.051
Mean (SD)	19.4 (13.1)	25.0 (27.4)	23.9 (25.4)		
Median	17.0	23.0	22.0		
Exercise min/week				3.18	.001*
Mean (SD)	70.0 (98.6)	118.1 (107.8)	108.9 (107.6)		
Median	30.0	105.0	90.0		
Alcohol drinks/week				0.91	.361
Mean (SD)	2.6 (4.5)	3.1 (3.9)	3.0 (4.0)		
Median	1.0	2.0	2.0		

Z: standardized test statistics for Mann-Whitney *U*.

* Indicates a significant *P*-value at an alpha of .05.

**Table 2.** Chi Square and Fisher’s Exact Comparisons on Physicians’ Self-Perceived Satisfaction With Taking Care of Own Health.

Variable	n (%)			*χ*²	*P*
	Dissatisfied (n = 43)	Satisfied (n = 182)	Total (N = 225)		
Gender				0.45	.581
Male	32 (74.4)	126 (69.2)	158 (70.2)		
Female	11 (26.5)	56 (30.8)	67 (29.8)		
Age				5.56	.061
<35-44	18 (41.9)	45 (24.7)	63 (28.0)		
45-54	11 (25.6)	49 (26.9)	60 (26.7)		
55 to >65	14 (32.6)	88 (48.4)	102 (45.3)		
Specialty				0.43	.511
Family medicine	22 (51.2)	83 (45.6)	105 (46.7)		
Other	21 (48.8)	99 (54.4)	120 (53.3)		
Graduate medical school				5.91	.016*
Canada	33 (76.7)	103 (56.6)	136 (60.4)		
Foreign	10 (23.3)	79 (43.4)	89 (39.6)		
Completed residency				2.30	.172
Canada	36 (83.7)	132 (72.5)	168 (74.7)		
Foreign	7 (16.3)	50 (27.5)	57 (25.3)		

* Indicates a significant *P*-value at an alpha of .05.

**Table 3.** Chi Square and Fisher’s Exact Comparisons of Lifestyle and Personal Health on Physicians’ Self-Perceived Satisfaction With Taking Care of Own Health.

Variable	n (%)			χ²	*P*
	Dissatisfied (n = 43)	Satisfied (n = 182)	Total (N = 225)		
General health				47.7^¶^	<.001*
Poor/fair	11 (25.6)	1 (0.5)	12 (5.3)		
Good/very good	31 (72.1)	124 (68.1)	155 (68.9)		
Excellent	1 (2.3)	57 (31.3)	58 (25.8)		
Regular GP				8.04	.007*
No	22 (51.2)	52 (28.6)	74 (32.9)		
Yes	21 (48.8)	130 (71.4)	151 (67.1)		
Self-prescribing				3.2^¶^	.361
Self	2 (4.7)	11 (6.0)	13 (5.8)		
Family member	12 (27.9)	52 (28.6)	64 (28.4)		
Both	13 (30.2)	33 (18.1)	46 (20.4)		
Neither	16 (37.2)	86 (47.3)	102 (45.3)		
Physical health affected workload				7.98	.007*
No	27 (62.8)	150 (82.4)	177 (78.7)		
Yes	16 (37.2)	32 (17.6)	48 (21.3)		
Last year hospitalization				2.64^¶^	.130
No	40 (93.0)	178 (97.8)	218 (96.9)		
Yes	3 (7.0)	4 (2.2)	7 (3.1)		
Co-morbidities				4.71	.034*
No	22 (51.2)	125 (68.7)	147 (65.3)		
Yes	21 (48.8)	57 (31.3)	78 (34.7)		
Smoking				5.53^¶^	.049*
No	40 (93.0)	180 (98.9)	220 (97.8)		
Yes	3 (7.0)	2 (1.1)	5 (2.2)		
Moderate exercise/week				10.13	.002*
No	20 (46.5)	41 (22.5)	61 (27.1)		
Yes	23 (53.5)	141 (77.5)	164 (72.9)		
Had colonoscopy				1.13	.311
No	27 (62.8)	98 (53.8)	125 (55.6)		
Yes	16 (37.2)	84 (46.2)	100 (44.4)		
Herpes Zoster vaccine				8.33^¶^	.015*
No	19 (44.2)	92 (50.5)	111 (49.3)		
Yes	2 (4.7)	33 (18.1)	35 (15.6)		
N/A	22 (51.2)	57 (80.9)	79 (35.1)		
Last GP appointment				7.53	.057
Never	7 (16.3)	16 (8.8)	23 (10.2)		
<1 year	10 (23.3)	82 (45.1)	92 (40.9)		
1 to <2 years	13 (30.2)	39 (21.4)	52 (23.1)		
2 or more years	13 (30.2)	45 (24.7)	58 (25.8)		
Last physical checkup				7.7	.052
Never	6 (14.0)	15 (8.2)	21 (9.3)		
<1 year	7 (16.3)	69 (37.9)	76 (33.8)		
1 to <2 years	14 (32.6)	42 (23.1)	56 (24.9)		
2 or more years	16 (37.2)	56 (30.8)	72 (32.0)		
Last flu vaccine				7.78^¶^	.208
Never	3 (7.0)	8 (4.4)	11 (4.9)		
<1 year	33 (76.7)	146 (80.2)	179 (79.6)		
1 to <2 years	4 (9.3)	21 (11.5)	25 (11.1)		
2 or more years	3 (7.0)	7 (3.8)	10 (4.4)		
Last measured cholesterol				13.03^¶^	.025
Never	4 (9.3)	7 (3.8)	11 (4.9)		
<1 year	17 (39.5)	98 (53.8)	115 (51.1)		
1 to <2 years	10 (23.3)	49 (26.9)	59 (26.2)		
2 or more years	12 (27.9)	28 (15.4)	40 (17.8)		
Last fasting blood sugar/A1C				4.04^¶^	.256
Never	4 (9.3)	15 (8.2)	19 (8.4)		
<1 year	17 (39.5)	96 (52.7)	113 (50.2)		
1 to <2 years	10 (23.3)	42 (23.1)	52 (23.1)		
2 or more years	12 (27.9)	29 (15.9)	41 (18.2)		

* Indicates a significant *P*-value at an alpha of .05.

¶ Indicates a Fisher’s Exact chi square is being reported.

### Physicians’ Self-Perceived Satisfaction With Taking Care of Own Health

Tables 1 to 3 display the unadjusted comparisons of preventive health measures and physician characteristics as they relate to participants’ self-perceived satisfaction with self-care. The unadjusted results suggest that, compared to those who were satisfied with their own health, physicians who were dissatisfied with how they take care of their own health had exercised less minutes per week (mean: 70 vs 118), and were graduates from Canadian medical schools (Tables 1 and 2). Further, lifestyle and personal health comparisons suggested that the majority of participants who were dissatisfied with their own self-care rated their health as poor/fair (25.6% vs 0.5%), did not have a regular GP (51.2% vs 28.6%), their workload was affected by their physical health in the past 4 weeks (37.2% vs 17.6%), had comorbidities (48.8% vs 31.3%), had not had the herpes zoster vaccine and were smokers compared to those who were satisfied (Table 3). The last 2 variables were deemed not relevant given their very low incidence and were thus omitted in the logistic regression analysis.

The results displayed in Table 4 indicate that 5 variables were independent predictors of physicians’ self-perceived satisfaction with taking care of own health. Physicians who graduated from Canadian medical schools (OR = 0.35; 95% CI 0.15-0.80), had comorbidities (OR = 0.31; 95% CI 0.13-0.72), and were between the age of 45 and 54 years (OR = 0.39; 95% CI 0.17-0.92) were less likely to be satisfied with taking care of their health. Conversely, physicians who had a regular GP (OR = 2.43; 95% CI 1.12-5.52) and performed moderate exercise per week (OR = 2.28; 95% CI 1.05-4.94) were more than 2 times likely to be satisfied with taking care of their own health.

**Table 4.** Independent Factors Associated With Satisfaction With Taking Care of Own Health After Logistic Regression Model (N = 225).

Variables	β	SE	*P*	OR	95% CI
Age 45-54	−.94	0.45	.031	0.39	0.17-0.92
Graduated Medical Education (Canada)	−1.04	0.42	.013	0.35	0.15-0.80
Had regular GP	.89	0.39	.024	2.43	1.12-5.52
Co-morbidities	−1.18	0.43	.006	0.31	0.13-0.72
Moderate exercise/week	.82	0.40	.037	2.28	1.05-4.94

Table 5 compares data on the preventive health measures from our sample with the Canadian physician national statistics. Overall, sample characteristics in our study were similar to those of other Canadian physicians. The respondents were less likely to be women, but in similar ratio to the rest of the nation (29.8% vs 34%). Moreover, the proportion of family doctors (46.7% vs 45%), as well as the total working hours per week (mean: 49.7 h vs 48 h), were also comparable. However, a higher proportion of Windsor-Essex physicians were trained outside of Canada (39.6% vs 21%) and were over the age of 45 years (72% vs 67%).

**Table 5.** Clinical Preventive Health Measures Undertaken by Physician Respondents at Time of Survey, Contrasted With Those of the 2009 Health Practices of Canadian Physicians Study by Frank and Segura (N = 225).

Variable	Last time performed [n (%)]						
	<1 year	1 to <2 years	2 to <3 years	3 to <4 years	4 to <5 years	≥5 years	Never
Appointment with doctor							
Current study	92 (40.9)	52 (23.1)	12 (5.3)	9 (4.0)	10 (4.4)	27 (12.0)	23 (10.2)
National data	NA	NA	NA	NA	NA	NA	NA
Physical check-up							
Current study	76 (33.8)	56 (24.9)	17 (7.6)	13 (5.8)	9 (4.0)	33 (14.7)	21 (9.3)
National data	(39)	(22)	(9)	(4)	(3)	(16)	(7)
Blood pressure check							
Current study	153 (68)	43 (19.1)	11 (4.9)	4 (1.8)	1 (0.4)	9 (4.0)	4 (1.8)
National data	(67)	(17)	(6)	(3)	(2)	(5)	(1)
Flu vaccine							
Current study	179 (79.6)	25 (11.1)	6 (2.7)	1 (0.4)	2 (0.9)	1 (0.4)	11 (4.9)
National data	(75)	(10)	(3)	(1)	(1)	(2)	(8)
Cholesterol measurement							
Current study	115 (51.1)	59 (26.2)	16 (7.1)	5 (2.2)	5 (2.2)	14 (6.2)	11 (4.9)
National data	(44)	(21)	(11)	(5)	(3)	(7)	(10)
Fasting blood sugar/A1c							
Current study	113 (50.2)	52 (23.1)	17 (7.6)	7 (3.1)	5 (2.2)	12 (5.3)	19 (8.4)
National data	NA	NA	NA	NA	NA	NA	NA
Hemoccult test (FOBT)							
Current study	32 (14.2)	18 (8.0)	12 (5.3)	4 (1.8)	5 (2.2)	10 (4.4)	144 (64.0)
National data	NA	NA	NA	NA	NA	NA	NA
PSA (men)							
Current study	68 (43.0)	24 (15.2)	4 (2.5)	4 (2.5)	2 (1.3)	6 (3.8)	50 (31.6)
National data	NA	NA	NA	NA	NA	NA	NA
Mammogram (women)							
Current study	10 (14.9)	15 (22.4)	7 (10.4)	3 (4.5)	2 (3.0)	0 (0.0)	30 (44.8)
National data	(26)	(16)	(5)	(2)	(2)	(4)	(45)
Pap smear (women)							
Current study	14 (20.9)	19 (28.4)	14 (20.9)	7 (10.4)	4 (6.0)	6 (9.0)	3 (4.5)
National data	(47)	(29)	(11)	(4)	(2)	(6)	(2)

**Figure 1.**
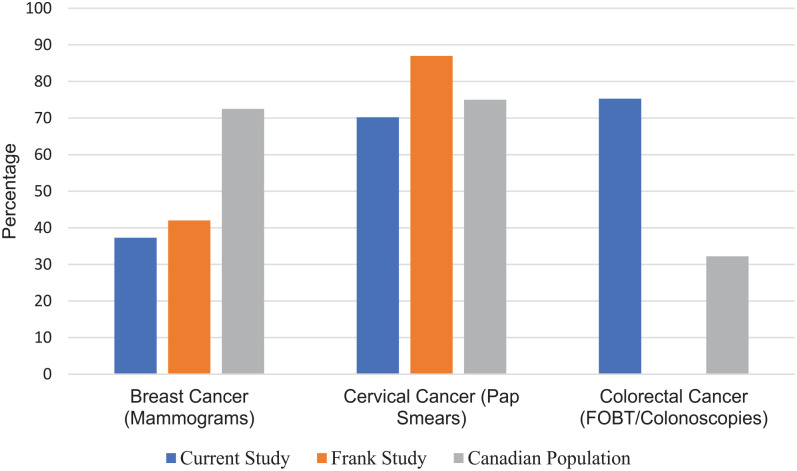
Cancer screening compliance. Cancer screening compliance undertaken by physician respondents at time of survey, compared with the 2009 Health Practices of Canadian Physicians study by Frank and Segura, and the general Canadian population. (N = 225).

Figure 1 compares cancer screening compliance of physicians in our study to that of the national physician study and general public statistics. Compared to the general public, compliance with cancer screening guidelines was below average for cervical (70.2% vs 75%) and breast cancer (37.3% vs. 72.5%) but above average for colon cancer (77.8% vs 32.2%).

## Discussion

Physicians in our region compared well with their national counterparts with regards to some important physical health characteristics. More than 94.7% of the respondents reported being in at least good health. This proportion is close to the 92% of Canadian physicians and much better than the 60% of the general population.^10,18^ Moreover, physicians in our region were substantially less likely to report smoking and alcohol consumption than their national counterparts. Only 2.2% of the physicians in our study were currently smoking compared to the 3.3% of Canadian physicians and 21% of the general population.^10,18^ Only 0.4% of participants in our study reported alcohol consumption of more than 5 drinks in 1 occasion weekly, compared to 2% of Canadian physicians.^10^ While 94.7% of respondents in our study reported being in at least good health, only 81% were satisfied with how well they take care of themselves.

One important objective of our study was to explore the factors associated with satisfaction with taking care of one’s health. Table 4 indicates that there were some positive and negative predictors of overall health satisfaction. Having a regular physician and engaging in regular moderate exercise were strong positive predictors of satisfaction. Almost 33% of the physicians reported that they do not have a regular family physician, which is significantly higher than the 17% of the local general population who do not have access to a regular family physician, as verified by the Windsor-Essex Regional Physician Recruitment Office. Moreover, 27.1% of respondents in our study did not engage regularly in at least moderate exercise; those who reported that they exercised did so in an average of 109 min/week of moderate to strenuous activity. This average time is much less than the 162 min/week of Canadian physicians and the 150 min/week recommended in the Canadian Physical Activity Guideline.^19^ Interestingly, being a Canadian medical graduate was a negative predictor of overall health satisfaction. We are not aware of any studies comparing the health of Canadian medical graduates to their international counterparts. Therefore, it is difficult to explain the reasons for this finding. Additional studies may be warranted to further assess how region of medical graduation impacts a physician’s health outcomes.

Self-reports of our participants suggested that compliance with the Canadian Task Force on Preventive Health Care’s (CTFPHC’s) guidelines was varied among the 3 cancer screening guidelines examined. For colorectal cancer, as displayed in Table 5, 77.8% of individuals over the age of 55 were up to date with the screening guidelines. This is much higher than the 32.2% of the general Canadian population and 44.7% of the Ontario population who report having a screening test.^20^ Of note, fecal occult blood test (FOBT) rates were used in colon cancer screening compliance instead of Fecal Immunochemical Test (FIT) rates. The latter was not part of Cancer Care Ontario program at the time our study was designed. For cervical and breast cancer screening, female physicians were lagging behind the general population and other Canadian physicians. Only 70.2% of physicians in our study reported having the Papanicolaou test in the past 3 years, which was less than the 75% of Canadian women in the general public and 87% of all Canadian female physicians.^10^ Only 37.3% of female physicians reported having a mammogram in the last 2 years, compared to 72.5% of other Canadian women and 42% of other Canadian female physicians.^10,21^ Future research exploring the factors affecting compliance with cancer screening among female physician colleagues may be warranted.

Our results also suggest that most respondents (82.4%) agreed that a physician’s own adherence to good health practices positively impacts patient’s encouragement to make positive health choices. This finding is promising given that other studies have suggested a high correlation between physicians’ healthy personal habits and their ability to counsel their patients on related prevention issues.^22^ If physicians are more self-aware of their own adherence to a healthy lifestyle and its impact on patients, they may be more motivating than others.^23^ Future studies looking into the direct impact of physicians’ health habits on patient’s compliance with health guidelines are warranted.

Given the self-report nature of our data, we cannot rule out the possibility of response bias despite it being anonymous. That is, respondents may tend to provide socially desirable answers that may under or overestimate the true behavior of a study respondent. Also, it may be difficult to ascertain whether those who declined participation in the study were different from those who completed the survey. Our response rate of 36.6%, although not high, it is reasonable considering that it is comparable to the response rate of 40.5% that was reported in the national study.^10^ Nevertheless, our study provides important information about many personal and professional health characteristics of physicians from small to medium sized communities, like Windsor-Essex.

## Conclusion

In conclusion, our findings provide important insight into the region specific health needs of physicians. Despite having some striking similarities when compared to their national counterparts across Canada, physicians in our region are diverse in their strengths and weakness when it comes to taking care of their own health. These findings support the need for region specific formulation of interventions to reduce negative disparities in physician health, such as lack of exercise, low compliance with breast/cervical cancer screening as well as low access to primary care for physicians themselves as in the case of physicians in our study. Such targeted measures will not only help improve the health of Canadian physicians as a whole, but may also positively impact the care they provide to their patients.

## References

[1] Shanafelt TD Bradley KA Wipf JE Back AL. Burnout and self-reported patient care in an internal medicine residency program. Ann Intern Med. 2002;136(5):358-367. 11874308 10.7326/0003-4819-136-5-200203050-00008

[2] Shanafelt TD West C Zhao X , et al. Relationship between increased personal well-being and enhanced empathy among internal medicine residents. J Gen Intern Med. 2005;20(7): 559-564. 16050855 10.1111/j.1525-1497.2005.0108.x PMC1490167

[3] Wallace JE Lemaire JB Ghali WA. Physician wellness: a missing quality indicator. Lancet. 2009;374(9702):1714-1721. 19914516 10.1016/S0140-6736(09)61424-0

[4] Hartwig B Nichols A. GP Health & Well-Being: The Issues Explored. Brisbane North Division of General Practice; 2000.

[5] Kay MP Mitchell GK Del Mar CB. Doctors do not adequately look after their own physical health. Med J Aust. 2004;181(7):368-370. 15462653 10.5694/j.1326-5377.2004.tb06329.x

[6] Davidson SK Schattner PL. Doctors’ health-seeking behaviour: a questionnaire survey. Med J Aust. 2003;179(6):302-305. 12964913 10.5694/j.1326-5377.2003.tb05552.x

[7] Canadian Medical Association. CMA Guide to Physician Health and Well-Being: Facts, Advice and Resources for Canadian Doctors. Canadian Medical Association; 2003.

[8] Pullen D Lonie CE Lyle DM Cam DE Doughty MV. Medical care of doctors. Med J Aust. 1995;162(9):481-484. 7746206 10.5694/j.1326-5377.1995.tb140011.x

[9] Úallacháin GN. Attitudes towards self-health care: a survey of GP trainees. Ir Med J. 2007;100(6):489-491. 17668680

[10] Frank E Segura C. Health practices of Canadian physicians. Can Fam Physician. 2009;55(8):810-811.e7. 19675268 PMC2726100

[11] McCall L Maher T Piterman L. Preventive health behaviour among general practitioners in Victoria. Aust Fam Physician. 1999;28(8):854-857. 10495543

[12] Wachtel TJ Wilcox VL Moulton AW Tammaro D Stein MD. Physicians’ utilization of health care. J Gen Intern Med. 1995;10(5):261-265. 7616335 10.1007/BF02599883

[13] Chambers R. Health and lifestyle of general practitioners and teachers. Occup Med. 1992;42(2):69-78. doi:10.1093/occmed/42.2.69 1606309

[14] Ritblado JR Pong RW. Geographic Distribution of Physicians in Canada. desLibris; 2005.

[15] Frank E Brogan DJ Mokdad AH Simoes EJ Kahn HS Greenberg RS. Health-related behaviors of women physicians vs other women in the United States. Arch Intern Med. 1998; 158(4):342-348. 9487231 10.1001/archinte.158.4.342

[16] Dubey V Mathew R Iglar K . 2010. Preventive Care Checklist Forms. The College of Family Physicians of Canada. Accessed March 20, 2014. https://www.cfpc.ca/en/resources/periodic-health-examination/preventive-care-checklist-forms

[17] Hosmer D Lemeshow S. Applied Logistic Regression. Wiley; 2000.

[18] Canada Year Book. 2011. Statistics-Canada. Accessed March 20, 2015. http://www.statcan.gc.ca/pub/11-402-x/2012000/pdf/health-sante-eng.pdf

[19] Canadian Society of Exercise Physiology. 2011. Canadian Physical Activity Guidelines. Accessed March 20, 2016. http://csepguidelines.ca/

[20] Canadian Cancer Society. 2011. Canadian Cancer Statistics. Accessed April 20, 2017. https://publications.gc.ca/collections/collection_2011/statcan/CS2-37-2011-eng.pdf

[21] Shields M Wilkins K. An Update on Mammography Use in Canada. Citeseer; 2009. 19813435

[22] Frank E . STUDENTJAMA. Physician Health and patient care. JAMA. 2004;291(5):637. 14762049 10.1001/jama.291.5.637

[23] Frank E Breyan J Elon L. Physician disclosure of healthy personal behaviors improves credibility and ability to motivate. Arch Fam Med. 2000;9(3):287-290. 10728118 10.1001/archfami.9.3.287

